# Supplementary material for: Rapid Internalization and Nuclear Translocation of CCL5 and CXCL4 in Endothelial Cells
Source: Int J Mol Sci. 2021 Jul 8;22(14):7332. doi: 10.3390/ijms22147332 (PMC8305033; doi:10.3390/ijms22147332)
Supplement: Supplementary file 1 [file ijms-22-07332-s001.zip › ijms-1162735 proof done supplementary.pdf]

International Journal of *Molecular Sciences*

Article

Rapid internalization and nuclear translocation of CCL5 and CXCL4 in endothelial cells

Annemiek Dickhout[1], Dawid M. Kaczor[1], Alexandra C.A. Heinzmann[1], Sanne L.N. Brouns[1], Johan W.M. Heemskerk[1], Marc A.M.J. van Zandvoort[1,2] and Rory R. Koenen[1,3]

[1] Department of Biochemistry, Cardiovascular Research Institute Maastricht, Maastricht University, Maastricht, the Netherlands. Department of Genetics and Cell Biology, Molecular Cell Biology, School for Oncology and Developmental Biology,
[2] Institute for Molecular Cardiovascular Research IMCAR, RWTH Aachen University, Germany
[3] Institute for Cardiovascular Prevention (IPEK), LMU Munich, Munich, Germany.
* Correspondence: Rory R. Koenen, PhD, CARIM, Maastricht University, PO Box 616, 6200MD Maastricht, The Netherlands, r.koenen@maastrichtuniversity.nl

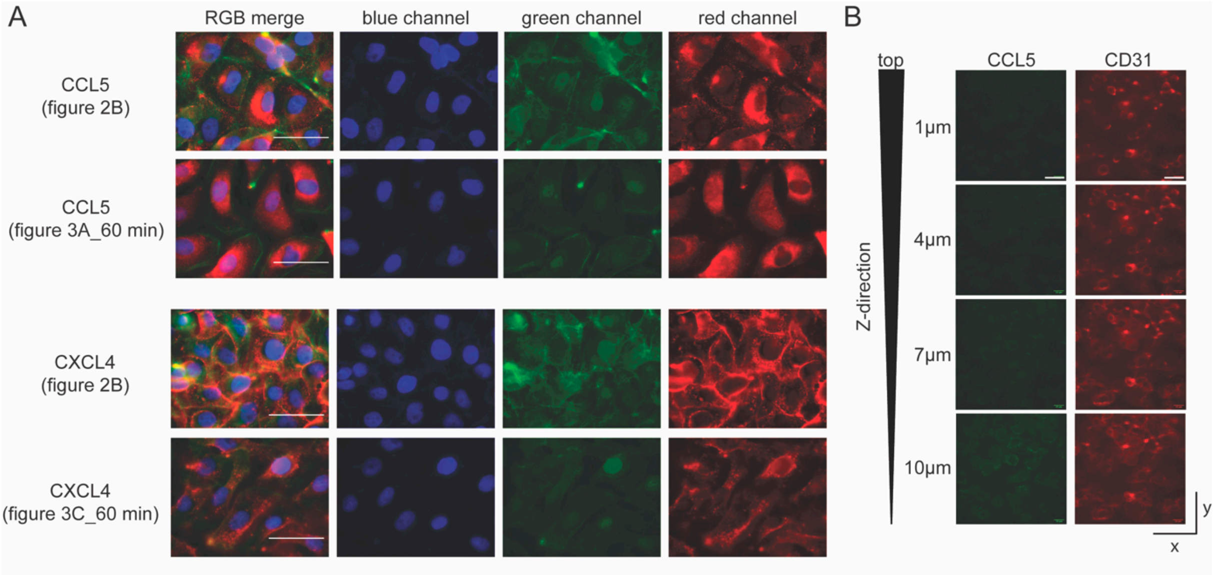

Figure S1. (A) Images of EAHy and chemokines split into individual colour channels. Indicated images from main figures 2 and 3 split into red, green and blue channels. Blue: nuclei (DAPI), green: actin (phalloidin-AF488), red: chemokine (CCL5 or CXCL4). Scale bar 50µm. (B) Fluorescence of cellular CCL5 (green) and CD31 (red) along the z-axis in EAHy at indicated relative distance, recorded by confocal microscopy after 60 minutes of incubation. Scale bar 50µm.

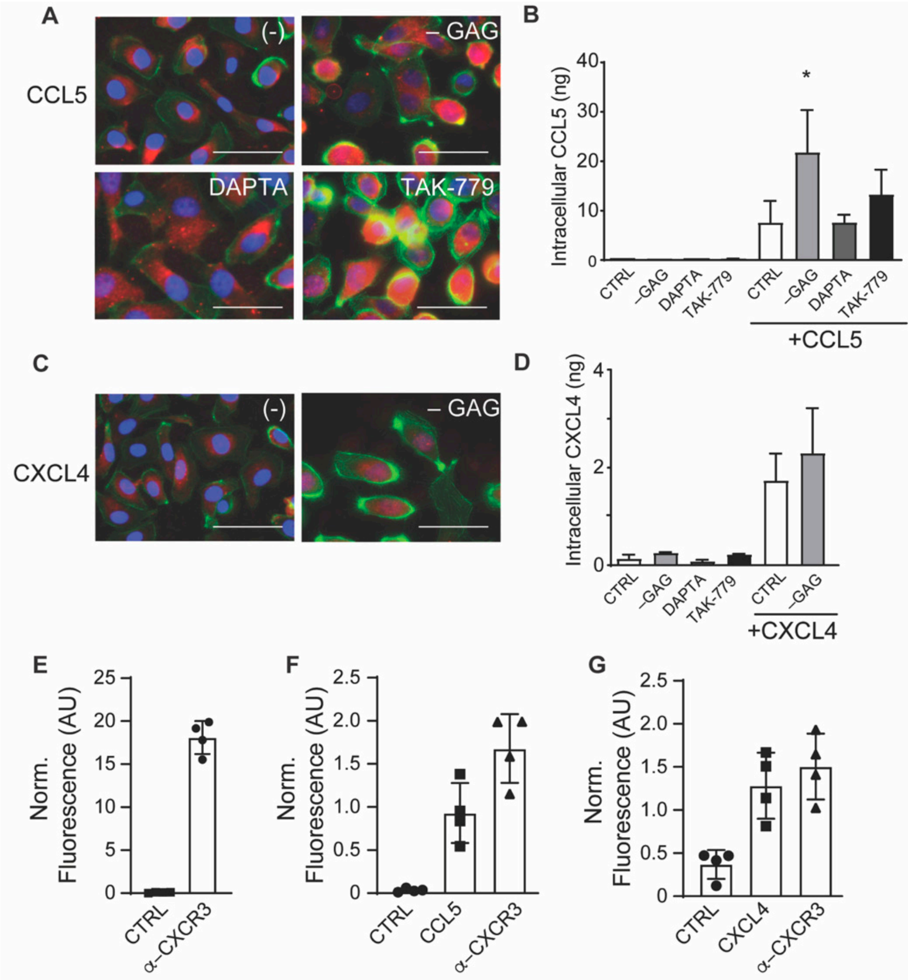

Figure S2. Internalization does not depend on GAGs or CXCR3 and is increased by an inhibitor of CCR5. EAHy were grown on a coverslip (A, C), in a 35 mm dish (B,D), or in a 96-well plate (E-F) and incubated with the enzymes heparinase III, chondroitinase ABC, hyaluronidase, and neuraminidase to cleave glycosaminoglycans (GAG), or DAPTA or TAK-779, inhibitors of CCR5 for 3 hours at 37°C. Blocking of CXCR3 was accomplished with antibodies for 15 minutes at 37°C. Cells were then incubated in presence of the inhibitors with CCL5 or CXCL4 for 60 minutes at 37 °C. Cells were washed with heparin prior to fixation and permeabilization(A, C, E, F, G) or lysis (B, D). (A, C) Cells were stained for the respective chemokine (red), F-actin (green), and nuclei (blue). Scale bar: 50 µm. (B, D) Quantification of CCL5 and CXCL4 levels using ELISA. (E, F, G) Cells were stained for the receptors, CCL5 or CXCL4 respectively, using Alexa Fluor 532, and nuclei (Hoechst). The cell count and fluorescence was analyzed with Cytation™. (n=6, $p<0.05$, Kruskal Wallis test).

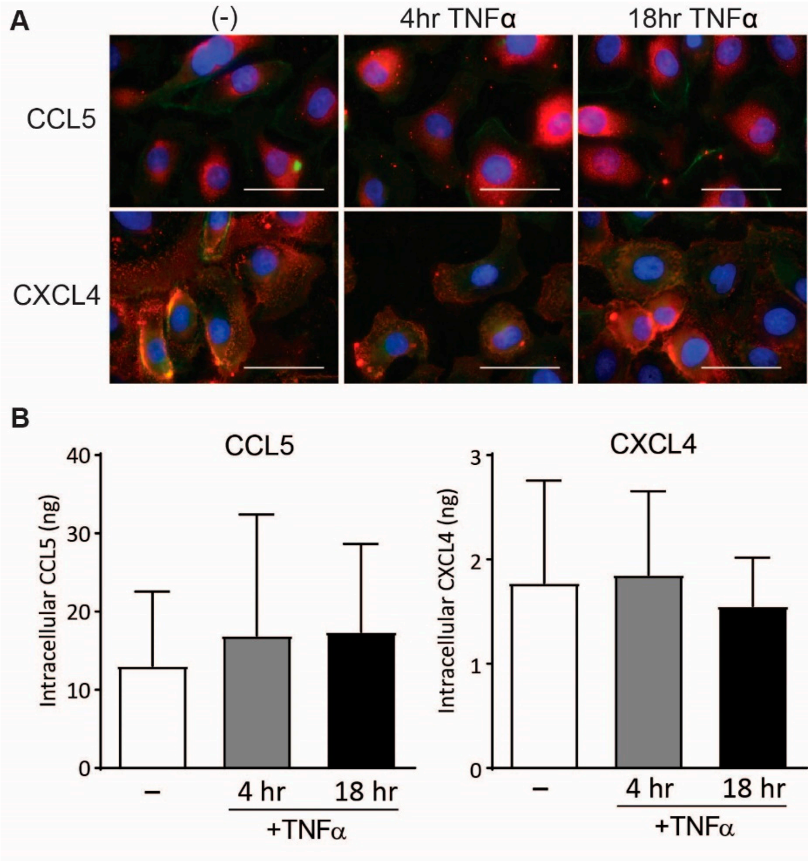

Figure S3. TNFα-treatment does not alter internalization of chemokines. EAHy cells were incubated with 10 ng/ml TNFα for 4 h or 18 h and were subsequently treated with the chemokines CCL5 (top row) or CXCL4 (bottom row) for 60 min 37 °C. (A) Cells were then washed with heparin prior to fixation and permeabilization and stained against CCL5 (top-red), CXCL4 (bottom-red), F-actin (green), and nuclei (blue). Scale bar: 50 µm (B) Cells were washed with heparin. Subsequently, CCL5 and CXCL4 levels in cell lysates were determined using ELISA. (n=6, ns, Kruskal Wallis).
